# Prognostic value of preoperative chemotherapy for thymic epithelial tumors: A propensity-matched analysis based on the SEER database

**DOI:** 10.3389/fsurg.2023.1108699

**Published:** 2023-03-17

**Authors:** Yan Fan, Tianjiao Cui, Shuai Wei, Xingcai Gao

**Affiliations:** ^1^ Department of Thoracic Surgery, The Fifth Affiliated Hospital of Zhengzhou University, Zhengzhou, China; ^2^ Department of Surgery, The First Affiliated Hospital of Zhengzhou University, Zhengzhou, China

**Keywords:** preoperative chemotherapy, chemotherapy, surveillance, epidemiology, end results program

## Abstract

**Background:**

The aim of this study was to assess the impact of preoperative chemotherapy on long-term survival (≥1 month) in patients with thymic epithelial tumors (TETs) and conditions suitable for chemotherapy using data from surveillance, epidemiology, and end-result databases.

**Methods:**

This retrospective study controlled for confounding factors by propensity score matching (PSM), analyzed overall survival (OS) and cancer-specific survival (CSS) by Kaplan-Meier methods, and analyzed factors affecting the prognosis of patients undergoing surgery for thymic epithelial tumors by univariate and multifactorial Cox regression.

**Results:**

A total of 2,451 patients who underwent surgery for TETs were identified from the Surveillance, Epidemiology, and End Results database. Preoperative chemotherapy significantly improved OS and CSS in patients with stage III/IV TETs compared to patients without preoperative chemotherapy. Subgroup analysis showed that patients younger than 60 years of age with TETs, patients with thymic carcinoma, and patients with TETs with multiple cancers were more likely to benefit from preoperative chemotherapy.

**Conclusion:**

This study found that preoperative chemotherapy is a viable option for advanced thymoma with favorable overall and cancer-specific survival rates, but patient history and physical condition should be fully considered in conjunction with diagnostic imaging findings to assess patient tolerance to chemotherapy.

## 1. Introduction

Thymic epithelial tumors (TETs) represent a group of heterogeneous, rare neoplasms arising from thymic epithelial cells and are the most common tumors of the anterior mediastinum (1). With disease progression, neoplastic cells invade mediastinal and thoracic organs, such as the lungs, heart, great vessels, surrounding nerves and lymph nodes, and may damage those organs (2). Thymic epithelial tumors include thymomas (Ts), thymic carcinomas (TCs) and thymic neuroendocrine tumors (NETs) (3, 4). Ts are classified into five types (A, AB, B1, B2, B3) in accordance with the shape and atypia of their epithelial cells as well as the abundance of lymphocytes (5).

The rarity of TETs, with an overall incidence of 0.13–0.32 per 100,000 people per year, has somewhat limited prospective studies, and optimal treatment options remain an unresolved issue (6). Studies on the effects of preoperative chemotherapy in patients with TETs are still inadequate, and the prognostic impact of chemotherapy on patients with TETs is still controversial and requires further study (7–11).

In this study, we aimed to assess the prognostic value of preoperative chemotherapy for TETs, its safety and its optimal conditions of application.

## 2. Methods

### 2.1. Patient selection

The Surveillance, Epidemiology, and End Results (SEER) database is one of the largest publicly available databases, with approximately 28% of the U.S. population covered (12). In this study, all cases were obtained from the SEER program (www.seer.cancer.gov), and patients from the SEER 17 registry (2007–2019; dataset submitted in November 2019) maintained by the National Cancer Institute were analyzed to extract patients with TETs using SEER*Stat software (version 8.4.0.1). Patients enrolled in this study were those with the ICD-O-3 site code C37.9 (thymus) and the ICD-O-3 histology codes thymoma (8580–8585), thymic carcinoma (8000, 8010, 8020, 8070, 8071, 8072, 8074, 8082, 8083, 8123, 8140, 8200, 8260, 8310, 8430, 8480, 8481, 8560, 8586, 8589) and thymic neuroendocrine tumor (8012, 8013, 8041, 8044, 8240, 8246, 8249 and 8574) (13–15).

We identified 3,555 cases according to the following admission criteria: (1) year of diagnosis from 2007 to 2019; (2) ICD-O-3 site code C37.9 (thymus); (3) pathologically confirmed TETs, not diagnosed by autopsy or death certificate; (4) patients with complete data on age at diagnosis, sex, stage, treatment, histology, vital status, or months of survival; (5) considering that surgery may lead to immediate death in the short term (16) and the lack of information on postoperative complications in the SEER database, patients surviving <1 month were excluded from this study, which focused on the impact of the treatment approach on long-term survival (≥1 month) in patients with thymic epithelial tumors.

**Figure 1:**
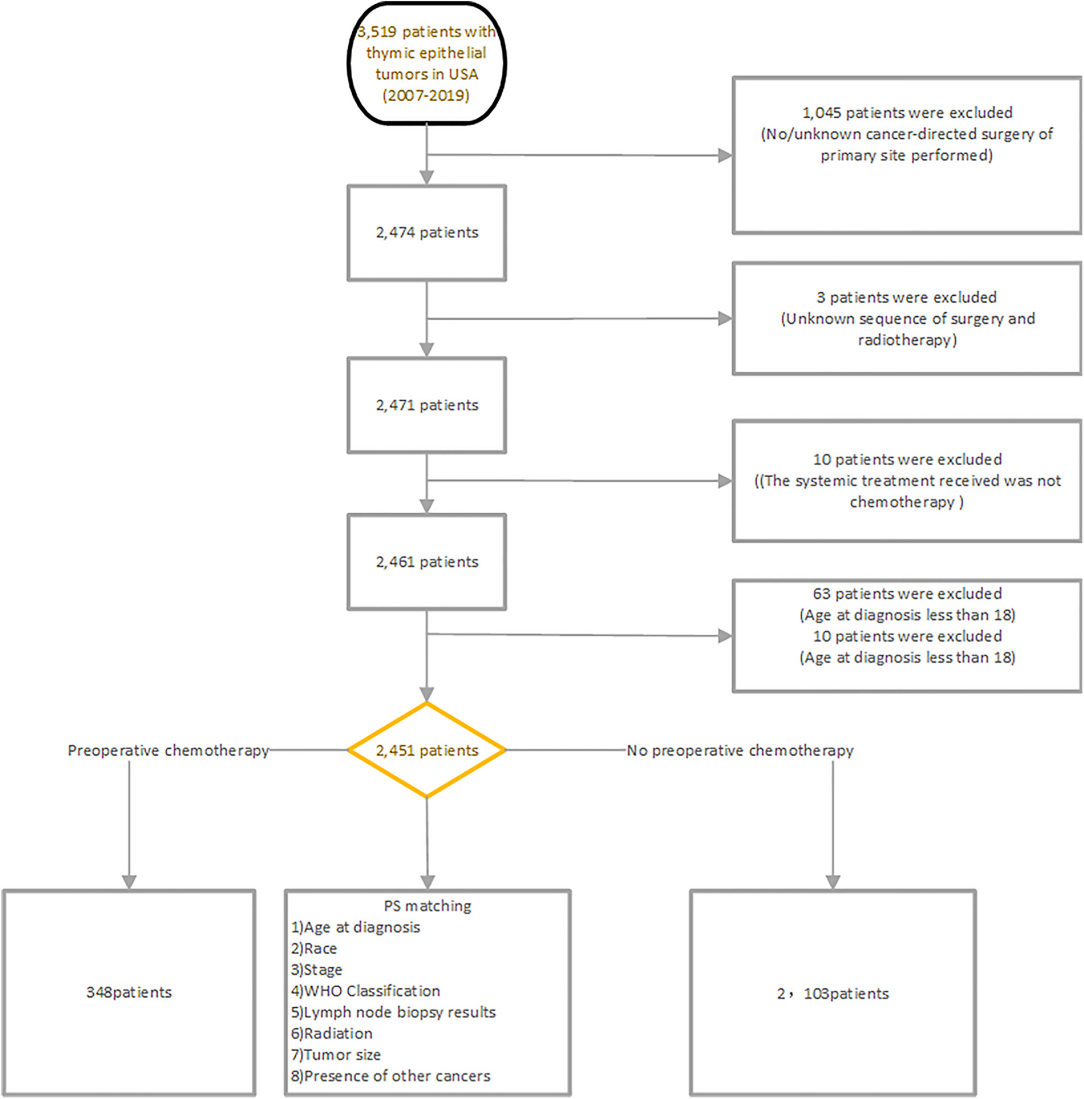
Demonstrates the flowchart of case inclusion and exclusion in detail.

For further study, patients who met the following criteria were excluded: (1) no/unknown cancer-directed surgery of primary site performed; (2) unknown sequence of surgery and radiotherapy; (3) the systemic treatment received was not chemotherapy; (4) age at diagnosis less than 18; (4) ethnicity information unknown Figure 1 demonstrates the flowchart of case inclusion and exclusion in detail.

This study used previously collected anonymized and de-identified data from the SEER database. Therefore, this study was exempted from ethical review by the Institutional Review Board of the Fifth Affiliated Hospital of Zhengzhou University.

### 2.2. Study Variables

The variables involved in our study included basic demographic information (age at diagnosis, sex, marital status, and race), neoplasm-related information: tumor size, WHO classification (A, AB, B1, B2, B3, TCs and NETs), Masaoka–Koga Stage (I/IIA, IIB, III/IV), Number of tumors (One primary tumor only, With other malignant tumors), and therapeutic information: Lymph node biopsy (Negative, Not performed, Positive); Radiotherapy Information (no_Radiotherapy, Radiotherapy after surgery, Radiotherapy prior to surgery);Chemotherapy (no_systematic treatment, Preoperative systemic treatment, Postoperative systemic treatment), survival information: survival months (from diagnosis to death or last follow-up), vital status (Live, Dead). Overall survival (OS) and cancer-specific survival (CSS) were the primary study endpoints. OS was defined as the time from diagnosis of TETs to death or loss to follow-up for any reason; CSS was defined as the time from the date of diagnosis to direct or indirect death from thymic epithelial tumors.

For two groups of continuity indicators (age and tumor size) X-tile software was used to select the best cut-off point in the survival data and to group the age and tumor size. The optimal groupings for age at diagnosis in this study were the <60 years group and the ≥60 years group; the optimal groupings for tumor size were the <80 mm group, the ≥80 mm group and the unknown size group. For marital status, patients were divided into a married group, a single (never married) group, and an other group. For race, patients were grouped into white group, black group, and Asian/Other ethnic group (including Pacific Islander, Alaska Native, etc.). The sequence of surgical vs. systemic treatment is recorded in the SEER database, in which systemic treatment mainly refers to chemotherapy, but also includes hormonal treatment, BRM treatment and transplant/endocrine cases. In this study, we included only cases where the type of systemic treatment was chemotherapy. The staging in the SEER database was divided into local, regional, and distal disease, and we reclassified the included patients with TETs into three groups according to the corresponding Masaoka–Koga staging as follows (the exact correspondence is indicated in Table 1): stage-I/IIA (Localized only), stage-IIB (Regional), and stage-III/IV (Distant site(s)/node(s) involved) (11, 17).

**Table 1:** Definition of Masaoka–Koga staging compared to staging groupings assigned using tumor data from SEER data.

Definition of Masaoka–Koga stage	The staging in the SEER database (17, 18)
I: Completely encapsulated tumors in the gross and microscopic	“Localized only”: “Invasive carcinoma confined to the primary gland” or “localized, not other specified”
IIA: Percutaneous invasion under microscope	
IIB: Gross invasion of the thymus or adjacent adipose tissue, or severe adhesions without breach of the mediastinal pleura or pericardium	“Regional”: “Tumor invades neighboring connective tissue”
III: Macroscopic invasion of adjacent organs (such as pericardium, great vessels, or lung parenchyma)	“Distant”: “neighboring organs/structures” or “further adjacent extensions” or “any positive lymph node”
IVA: Metastasis of the pleura or pericardium	
VB: Metastases of lymphatic or hematogenous origin	

### 2.3. Statistical analysis

This study was analyzed using R statistical software (www.r-project.org). Among patients who underwent surgery for TETs, Pearson *χ*^2^ tests or Fisher's exact tests were performed for patients who received different treatment modalities (no preoperative chemotherapy and preoperative chemotherapy) and treatment-related factors. Univariate and multifactorial Cox regression models were performed using “tableone”, “dplyr”, and “skimr” in R software to estimate hazard ratios (HR) and 95% confidence intervals (CI) to analyze independent prognostic factors associated with overall survival (OS) and cancer-specific survival (CSS) for patients undergoing TETs. Kaplan-Meier curves were plotted using the “Survival” package and “ggsurvplot” in R software to estimate OS and CSS for each group of patients, and *P* values were determined using the log-rank method. A 1 : 1 optimal nearest neighbor propensity score matching (PSM) was performed using the “MatchIt” package in R software to balance the baseline characteristics of patients in the study and control groups with a caliper value of 0.1. *P* < 0.05 was considered a statistically significant difference.

## 3. Results

### 3.1. Distribution Characteristic of Factors Related to Treatment Patterns

Our study enrolled 2,451 eligible patients who underwent surgery for TETs between 2007 and 2019. Among them, 1,686 were white, 346 were black, and 419 were patients of other ethnicities, including Pacific Islanders and Asians; The study included 1,747 patients with Ts, 576 patients with TCs, and 128 patients with NETs; Slightly more men than women were included in this study, with 53.7% of male patients compared to 46.3% of female patients.

Data retrievable in the study included age at diagnosis, race, sex, marital status, WHO classification, Masaoka–Koga stage, lymph node biopsy, sequence of radiation/chemotherapy vs. surgery, tumor size, and the presence of other tumors. The clinicopathological characteristics of patients with thymic epithelial tumor surgery before and after propensity score matching are presented in Table 2. Of the 2,451 patients with TETs, 348 received preoperative chemotherapy and 2,103 underwent direct surgery without preoperative chemotherapy. *χ*^2^ tests showed significant differences in the proportion of patients between the preoperative chemotherapy group and the no preoperative chemotherapy group at different levels of exposure variables (including Masaoka–Koga stage, tumor size, etc.). After PSM, the *P*-values for all covariates were above 0.1, indicating that all patient- and treatment-related factors were well balanced between the study and control groups. The results of the descriptive statistical analysis for the entire cohort and the matched cohorts are presented in Table 3.

**Table 2:** Clinicopathological characteristics of patients with thymic epithelial tumor surgery before and after propensity score matching.

Variables	Before propensity score matching				After propensity score matching			
	Total	no_POCT	POCT	*P*	Total	no_POCT	POCT	Total
*n*	2,451	2,103	348		614	307	307	
**Survival months [median (IQR)]**	50.00 [22.00, 90.00]	50.00 [22.00, 90.00]	46.50 [20.00, 89.00]	0.633	46.00 [19.00, 89.75]	46.00 [18.00, 90.00]	46.00 [20.00, 89.00]	0.505
**30-day mortality cases(30-day mortality)**	75	71 (3.38%)	4 (1.15%)		12	8 (2.61%)	4 (1.30%)	
**Age at diagnosis** [median (IQR)]	61.00 [51.00, 69.00]	62.00 [52.00, 70.00]	58.00 [46.00, 66.00]	<0.001	58.00 [46.00,66.75]	58.00 [46.00, 67.00]	58.00 [46.00, 66.00]	0.838
18–59 years	1,102 (45.0)	902 (42.9)	200 (57.5)	<0.001	345 (56.2)	170 (55.4)	175 (57.0)	0.745
60 + years	1,349 (55.0)	1,201 (57.1)	148 (42.5)		269 (43.8)	137 (44.6)	132 (43.0)	
**Tumor size**								
“<80 mm”	1,454 (59.3)	1,343 (63.9)	111 (31.9)	<0.001	207 (33.7)	102 (33.2)	105 (34.2)	0.803
“≥80mm”	815 (33.3)	611 (29.1)	204 (58.6)		342 (55.7)	170 (55.4)	172 (56.0)	
Unknown	182 (7.4)	149 (7.1)	33 (9.5)		65 (10.6)	35 (11.4)	30 (9.8)	
**Sex**								
Women	1,136 (46.3)	988 (47.0)	148 (42.5)	0.138	264 (43.0)	130 (42.3)	134 (43.6)	0.807
Male	1,315 (53.7)	1,115 (53.0)	200 (57.5)		350 (57.0)	177 (57.7)	173 (56.4)	
**Race**								
Black	346 (14.1)	299 (14.2)	47 (13.5)	0.089	81 (13.2)	40 (13.0)	41 (13.4)	0.573
Asians/Other Races	419 (17.1)	346 (16.5)	73 (21.0)		124 (20.2)	57 (18.6)	67 (21.8)	
White	1,686 (68.8)	1,458 (69.3)	228 (65.5)		409 (66.6)	210 (68.4)	199 (64.8)	
**WHO Classification**								
“Ts,A/AB”	550 (22.4)	495 (23.5)	55 (15.8)	0.005	99 (16.1)	49 (16.0)	50 (16.3)	0.237
“Ts,B1/B2”	571 (23.3)	493 (23.4)	78 (22.4)		151 (24.6)	81 (26.4)	70 (22.8)	
“Ts,B3”	307 (12.5)	254 (12.1)	53 (15.2)		90 (14.7)	46 (15.0)	44 (14.3)	
“Ts, NOS”	319 (13.0)	272 (12.9)	47 (13.5)		65 (10.6)	23 (7.5)	42 (13.7)	
NETs	128 (5.2)	114 (5.4)	14 (4.0)		31 (5.0)	17 (5.5)	14 (4.6)	
TCs	576 (23.5)	475 (22.6)	101 (29.0)		178 (29.0)	91 (29.6)	87 (28.3)	
**Masaoka–Koga stage**								
“I-IIA”	916 (37.4)	865 (41.1)	51 (14.7)	<0.001	83 (13.5)	38 (12.4)	45 (14.7)	0.443
“IIB”	1,118 (45.6)	949 (45.1)	169 (48.6)		328 (53.4)	174 (56.7)	154 (50.2)	
“III/IV”	356 (14.5)	233 (11.1)	123 (35.3)		193 (31.4)	90 (29.3)	103 (33.6)	
Unstaged	61 (2.5)	56 (2.7)	5 (1.4)		10 (1.6)	5 (1.6)	5 (1.6)	
**Radiotherapy**								
no radiotherapy	1,025 (41.8)	919 (43.7)	106 (30.5)	<0.001	263 (42.8)	129 (42.0)	134 (43.6)	0.874
PRRT	1,363 (55.6)	1,131 (53.8)	232 (66.7)		336 (54.7)	171 (55.7)	165 (53.7)	
PORT	63 (2.6)	53 (2.5)	10 (2.9)		15 (2.4)	7 (2.3)	8 (2.6)	
**With other tumors**								
NO	1,789 (73.0)	1,513 (71.9)	276 (79.3)	0.005	481 (78.3)	234 (76.2)	247 (80.5)	0.240
Yes	662 (27.0)	590 (28.1)	72 (20.7)		133 (21.7)	73 (23.8)	60 (19.5)	
**Lymph node biopsy**								
negative	890 (36.3)	737 (35.0)	153 (44.0)	<0.001	273 (44.5)	139 (45.3)	134 (43.6)	0.613
not performed	1,379 (56.3)	1,230 (58.5)	149 (42.8)		253 (41.2)	121 (39.4)	132 (43.0)	
positive	182 (7.4)	136 (6.5)	46 (13.2)		88 (14.3)	47 (15.3)	41 (13.4)	
**Marriage Status**								
Married	1,588 (64.8)	1,356 (64.5)	232 (66.7)	0.067	412 (67.1)	204 (66.4)	208 (67.8)	0.785
Other	433 (17.7)	386 (18.4)	47 (13.5)		88 (14.3)	47 (15.3)	41 (13.4)	
Single (never married)	430 (17.5)	361 (17.2)	69 (19.8)		114 (18.6)	56 (18.2)	58 (18.9)	

### 3.2. Identification of prognostic factors of OS and OSS

Table 4 lists the 11 variables included in the univariate Cox regression model to analyze the factors associated with overall survival or cancer-specific survival in patients undergoing surgery for TETs. Variables with univariate analysis *P* < 0.1 were enrolled in multivariate Cox Regression models. Multivariate Cox regression analysis demonstrated that preoperative chemotherapy was an independent prognostic factor for OS (*P* = 0.002) and CSS (*P* = 0.013) in patients undergoing surgery for TETs. In addition, age at diagnosis, Masaoka–Koga staging, WHO classification, radiotherapy and lymph node biopsy findings were all independent prognostic factors for both OS and CSS.

**Table 3:** Univariate and multifactorial analyses of propensity score matching affecting overall survival in patients undergoing surgery for thymic epithelial tumors.

Variables	OS						CSS					
	Univariate analysis			Multivariate analysis			Univariate analysis			Multivariate analysis		
	Hazard Ratio	95% CI	*P*	Hazard Ratio	95% CI	*P*	Hazard Ratio	95% CI	*P*	Hazard Ratio	95% CI	*P*
**Age**												
<60	1			1			1			1		
≥60	2.02	1.50–2.71	<0.001	1.96	1.42–2.69	<0.001	1.57	1.10–2.24	0.013	1.51	1.04–2.19	0.029
**Tumor size**												
“<80mm”	1						1					
“≥80 mm”	1.18	0.85–1.63	0.324				1.27	0.85–1.89	0.238			
Unknown	0.99	0.60–1.64	0.983				0.97	0.52–1.83	0.926			
**Race**												
Black	1						1					
Asians/Other Races	1.22	0.72–2.05	0.463				1.27	0.65–2.49	0.482			
White	1.13	0.72–1.79	0.591				1.35	0.75–2.42	0.316			
**Sex**												
Women	1						1					
Male	1.18	0.87–1.59	0.285				1.11	0.78–1.60	0.560			
**Masaoka–Koga stage**												
“I–IIA”	1			1			1			1		
“IIB”	1.73	0.94–3.16	0.076	1.51	0.81–2.83	0.197	1.59	0.75–3.34	0.224	1.26	0.58–2.74	0.552
“III/IV”	2.93	1.60–5.39	<0.001	2.71	1.43–5.13	0.002	3.29	1.57–6.89	0.002	2.59	1.19–5.61	0.016
Unstaged	1.32	0.37–4.69	0.666	1.48	0.40–5.40	0.554	1.41	0.30–6.62	0.667	1.41	0.29–6.82	0.671
**WHO Classification**												
“Ts,A/AB”	1			1			1			1		
“Ts,B1/B2”	1.66	0.87–3.14	0.124	1.74	0.90–3.37	0.101	2.22	0.89–5.53	0.087	2.11	0.83–5.34	0.115
“Ts,B3”	2.02	1.04–3.94	0.038	2.38	1.20–4.73	0.013	2.75	1.07–7.02	0.035	2.90	1.12–7.54	0.029
“Ts, NOS”	1.19	0.55–2.58	0.653	1.62	0.73–3.58	0.235	1.66	0.58–4.79	0.348	1.89	0.64–5.57	0.251
TCs	4.17	2.32–7.51	<0.001	4.10	2.21–7.59	<0.001	6.52	2.81–15.12	<0.001	5.68	2.38–13.57	<0.001
NETs	4.38	2.10–9.10	<0.001	3.48	1.59–7.66	0.002	8.30	3.19–21.61	<0.001	5.23	1.89–14.47	0.001
**Radiotherapy**												
no radiotherapy	1			1			1			1		
PRRT	0.93	0.41–2.13	0.861	0.60	0.25–1.43	0.249	1.20	0.48–3.00	0.692	0.67	0.26–1.75	0.415
PORT	0.71	0.53–0.95	0.023	0.51	0.38–0.70	<0.001	0.62	0.46–0.83	0.001	0.52	0.35–0.76	<0.001
**POCT**												
NO	1			1			1					
Yes	0.66	0.49–0.90	0.007	0.63	0.46–0.85	0.002	0.67	0.47–0.97	0.033	0.63	0.44–0.91	0.013
**With other tumors**												
NO	1						1					
Yes	1.12	0.80–1.56	0.504				0.96	0.63–1.47	0.852			
**Lymph node biopsy**												
negative	1						1			1		
not performed	1.03	0.73–1.44	0.879	1.15	0.81–1.62	0.427	1.24	0.81–1.91	0.324	1.37	0.89–2.13	0.157
positive	2.40	1.64–3.50	<0.001	1.61	1.06–2.45	0.027	3.54	2.25–5.56	<0.001	2.18	1.32–3.59	0.002
**Marriage Status**												
Married	1						1					
Other	1.13	0.76–1.67	0.536	1.07	0.72–1.60	0.735	0.92	0.55–1.52	0.737			
Single (never married)	0.67	0.44–1.03	0.069	0.99	0.63–1.56	0.965	0.71	0.43–1.18	0.181			

**Table 4:** Descriptive statistics for the entire cohort and matching cohort.

Variables	before propensity score matching				after propensity score matching			
	Mean	SD	Median	Min, Max	Mean	SD	Median	Min, Max
Survival months	58.20	42.81	50	1, 155	56.75	43.97	46	1, 155
Age at diagnosis	59.55	13.96	61	18, 92	55.80	14.54	58	19, 84
Tumor size (mm, Known size)	71.22	64.18	62	1, 989	92.11	78.46	82	1, 989

### 3.3. Survival analysis and Forest plots

**Figure 2:**
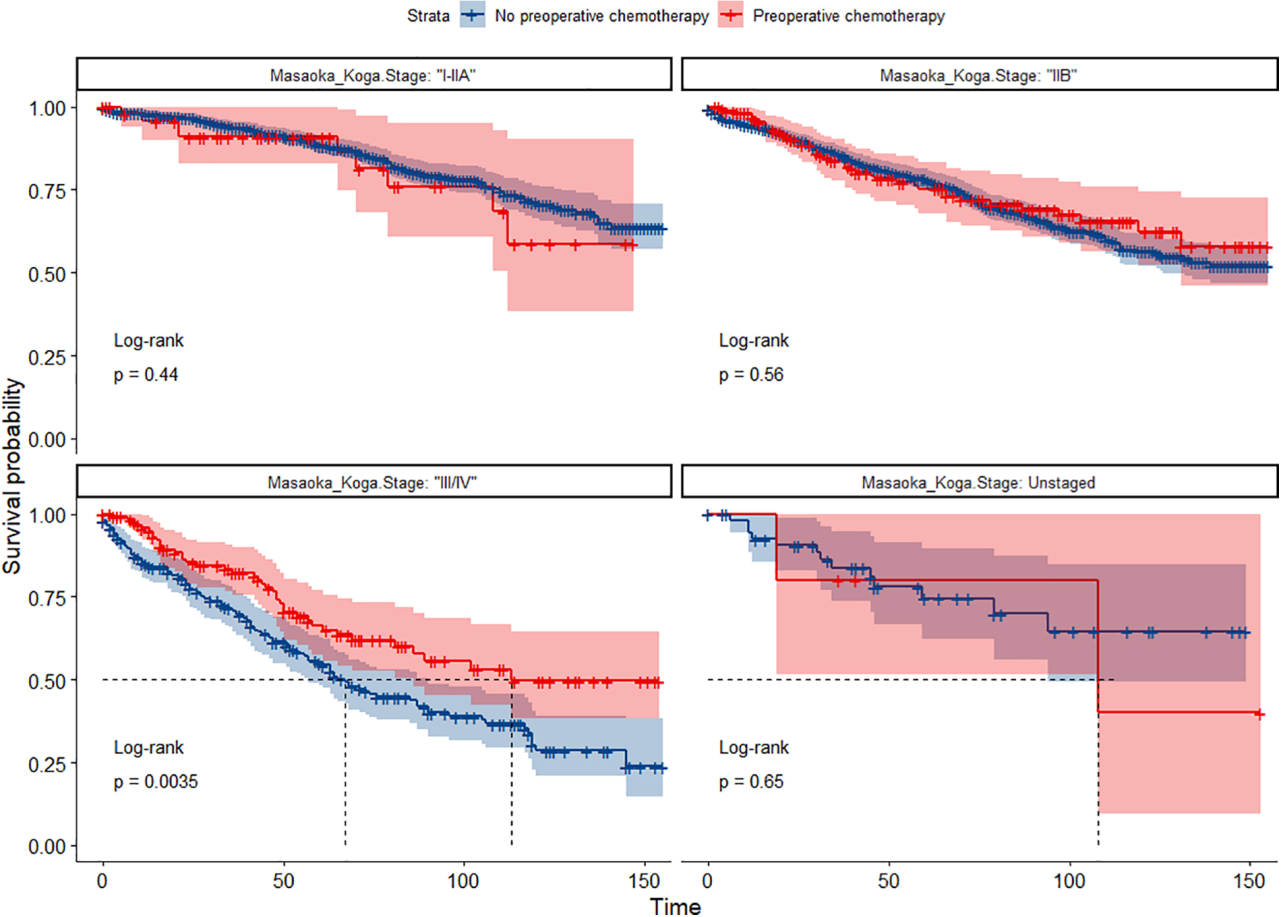
Survival curves of OS in the POCT group and no-POCT group in patients undergoing surgery for TETs at different stages before propensity matching.

**Figure 3:**
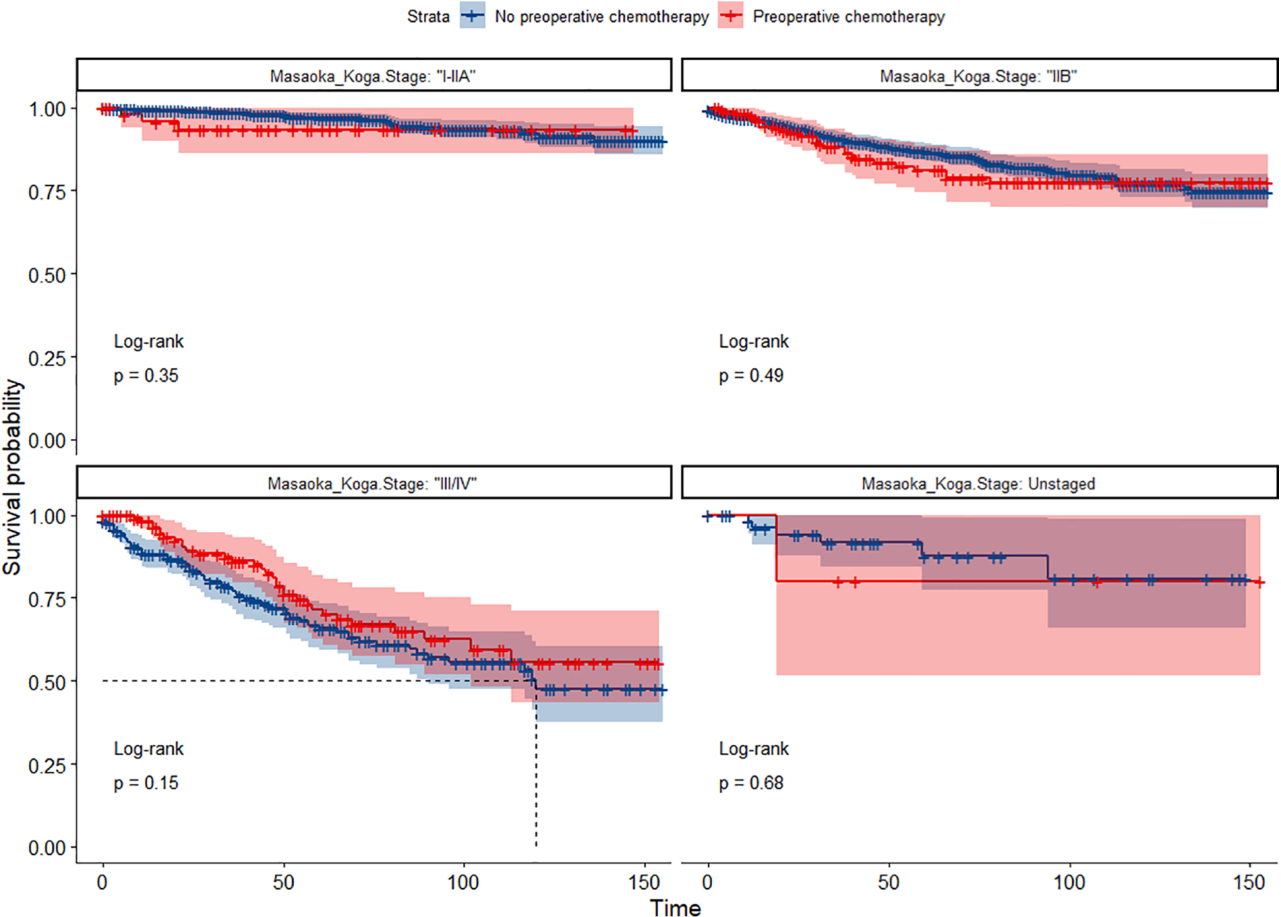
Survival curves of CSS in the POCT group and no-POCT group in patients undergoing surgery for TETs at different stages before propensity matching.

**Figure 4:**
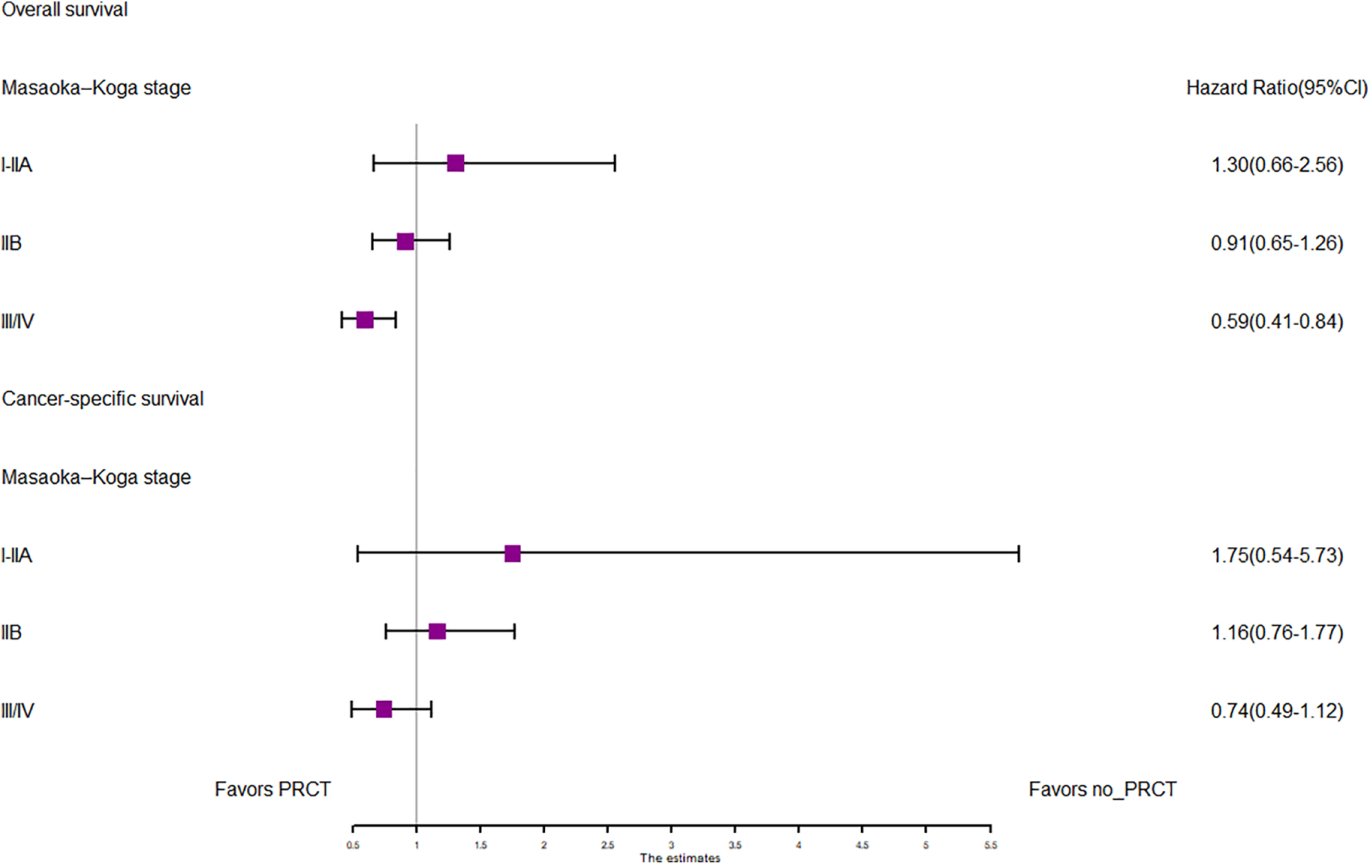
Forest plots of OS and CSS in the POCT and no POCT groups in patients undergoing surgery for TETs at different stages before propensity matching.

In the entire cohort before propensity score matching, Figures 2, 3 illustrate the prognosis of patients with TETs with different Masaoka–Koga staging who received preoperative chemotherapy vs. those who did not. Preoperative chemotherapy significantly improved OS (Median survival times in months, 102.72 vs. 79.67, *P *= 0.0035) in patients with stage III/IV and did not significantly improve OS or CSS in patients with stage I/IIA or IIB TETs (*P *> 0.05). Similarly, hazard ratios and 95% CIs for OS and CSS in patients with TETs of different Masaoka–Koga staging are shown in Figure 4, with preoperative chemotherapy being a favorable factor for OS in patients with III/IV.

**Figure 5:**
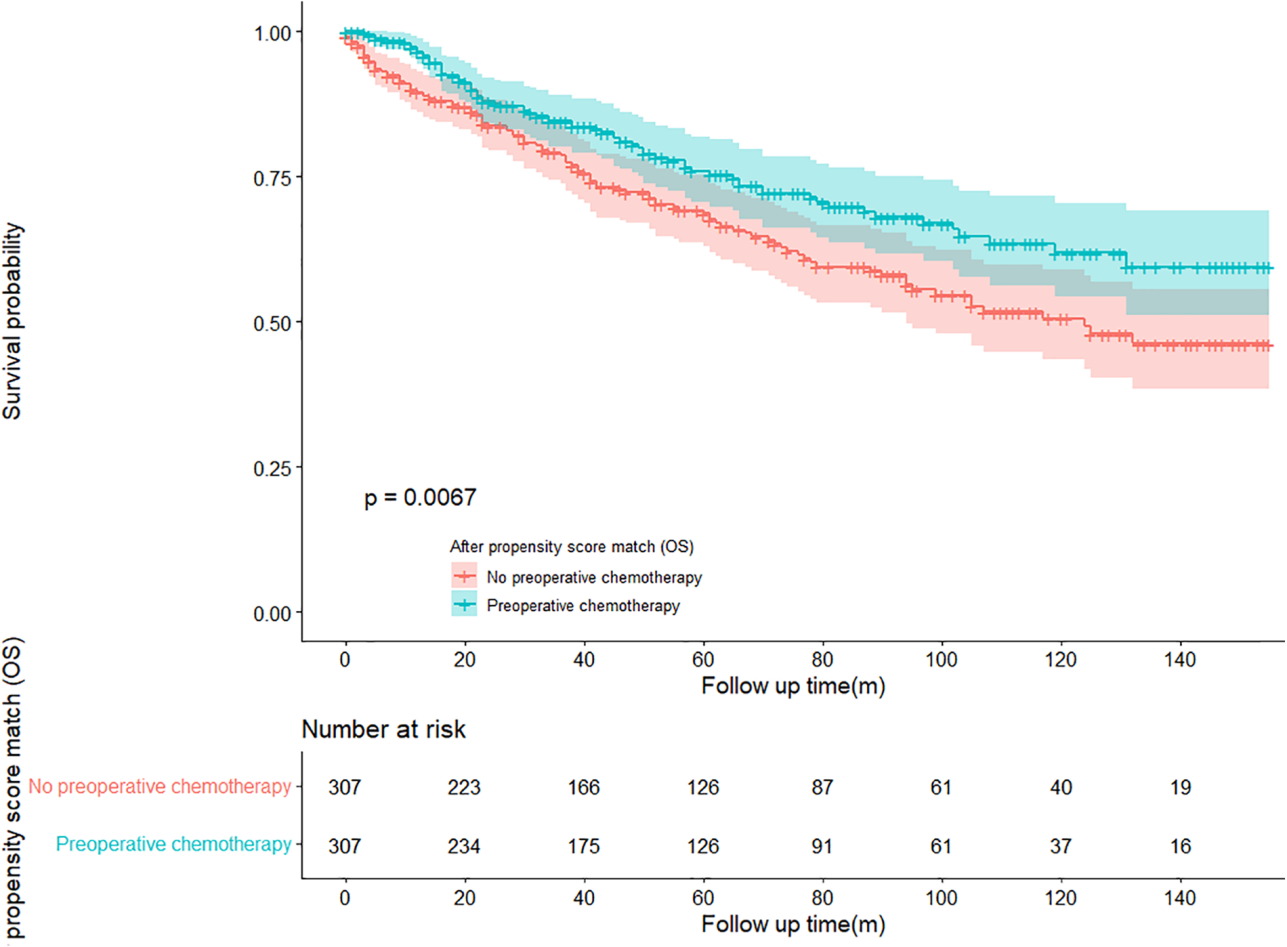
Survival curves of OS in the POCT group and no-POCT group in patients undergoing surgery for TETs after propensity matching.

**Figure 6:**
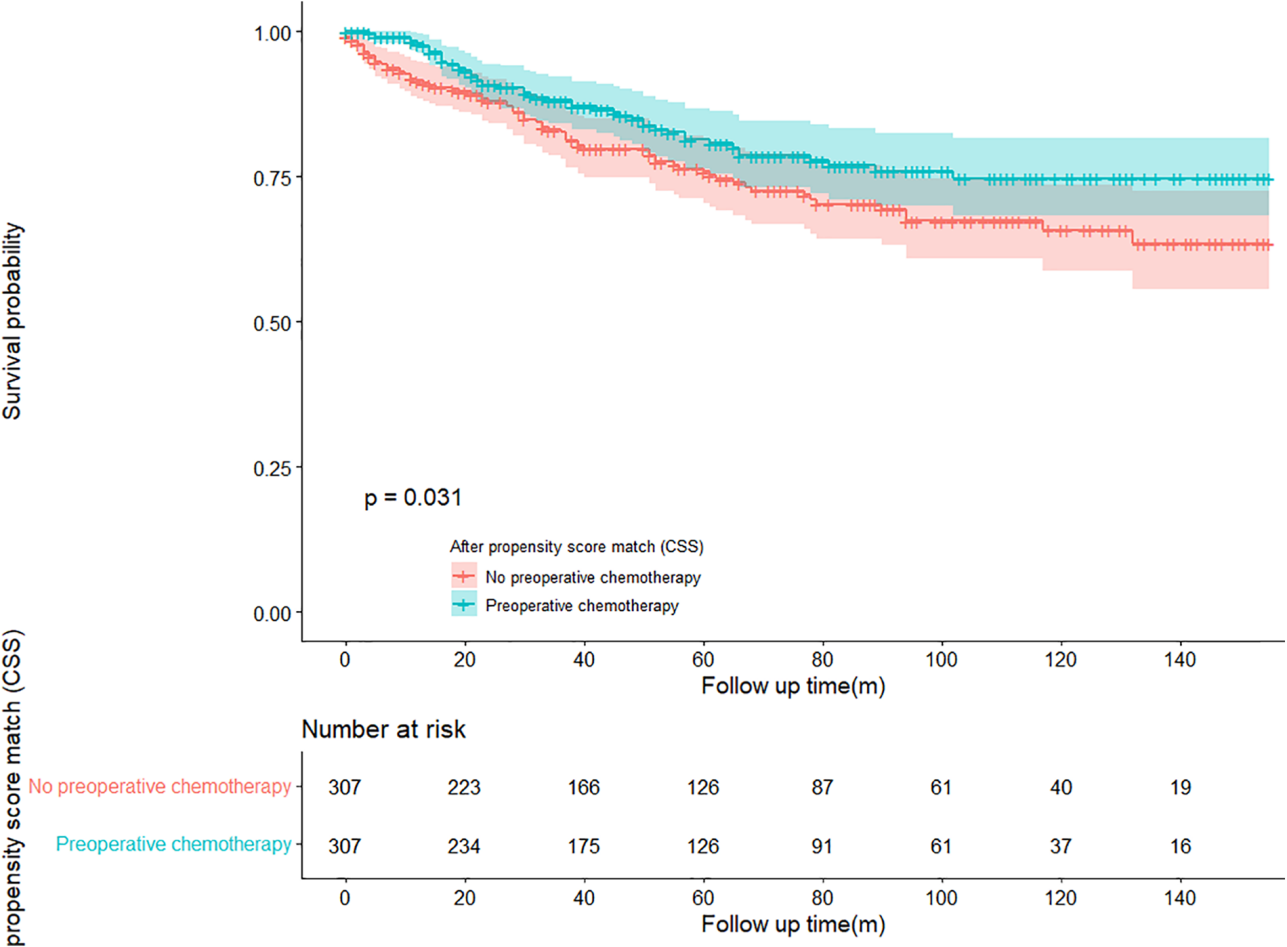
Survival curves of CSS in the POCT group and no-POCT group in patients undergoing surgery for TETs after propensity matching.

**Figure 7:**
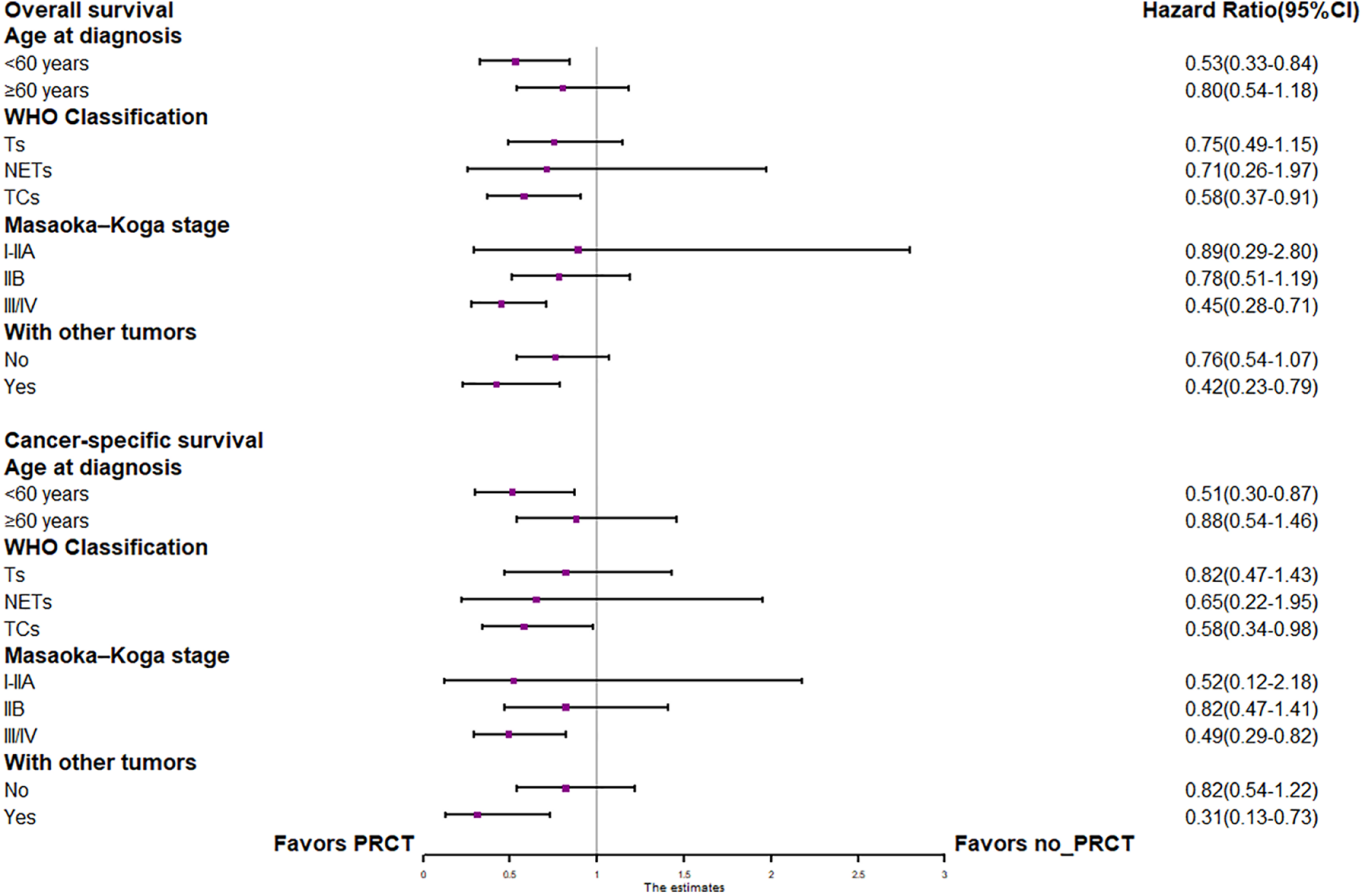
Forest plots of OS and CSS for subgroup analysis based on different grouping criteria.

**Figure 8:**
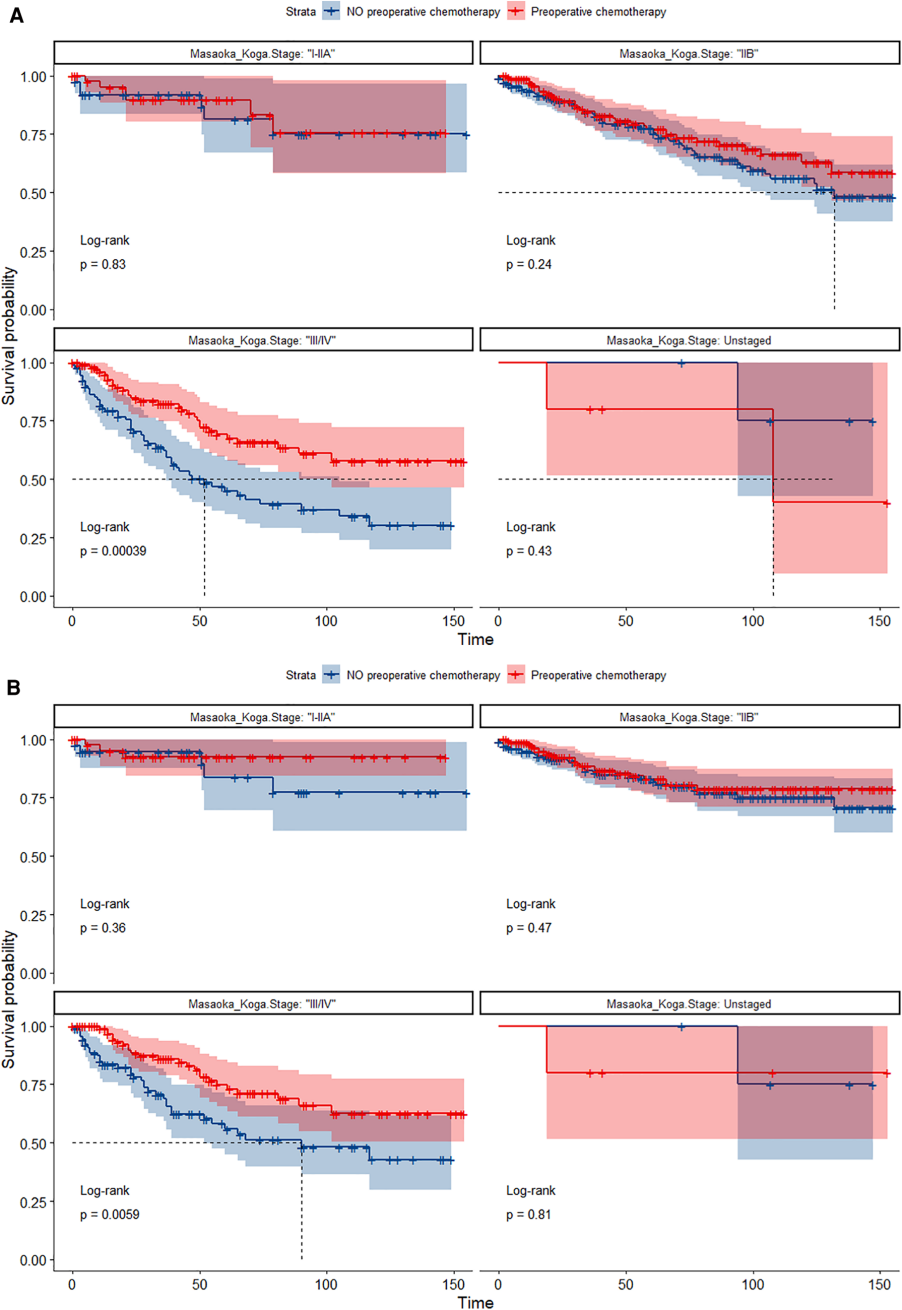
(**A**) Survival curves of OS in the POCT group and no-POCT group in patients undergoing surgery for TETs at different stages after propensity matching. (**B**) Survival curves of CSS in the POCT group and no-POCT group in patients undergoing surgery for TETs at different stages after propensity matching.

**Figure 9:**
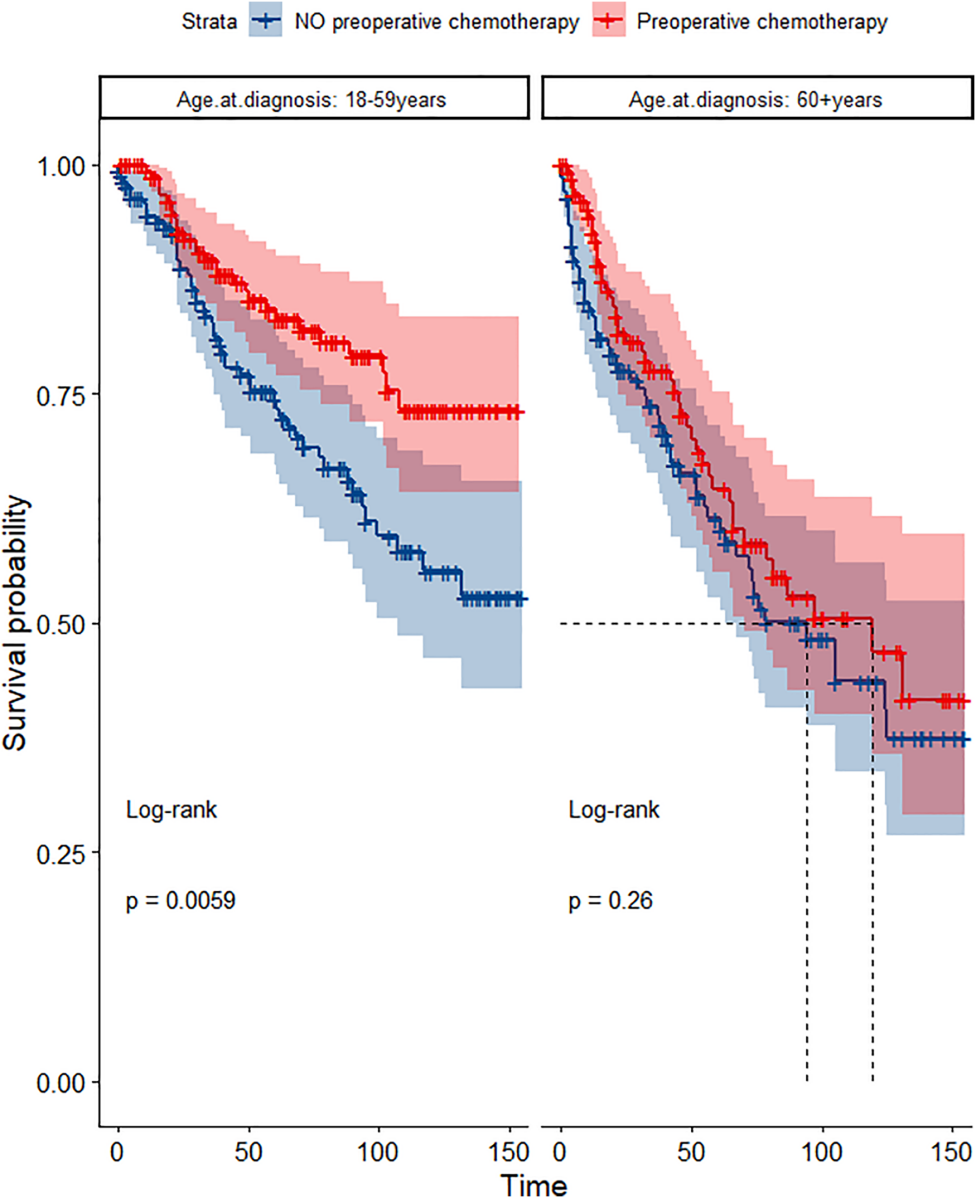
Survival curves of OS in the POCT and no POCT groups in patients undergoing surgery for TETs in different age groups after propensity matching.

**Figure 10:**
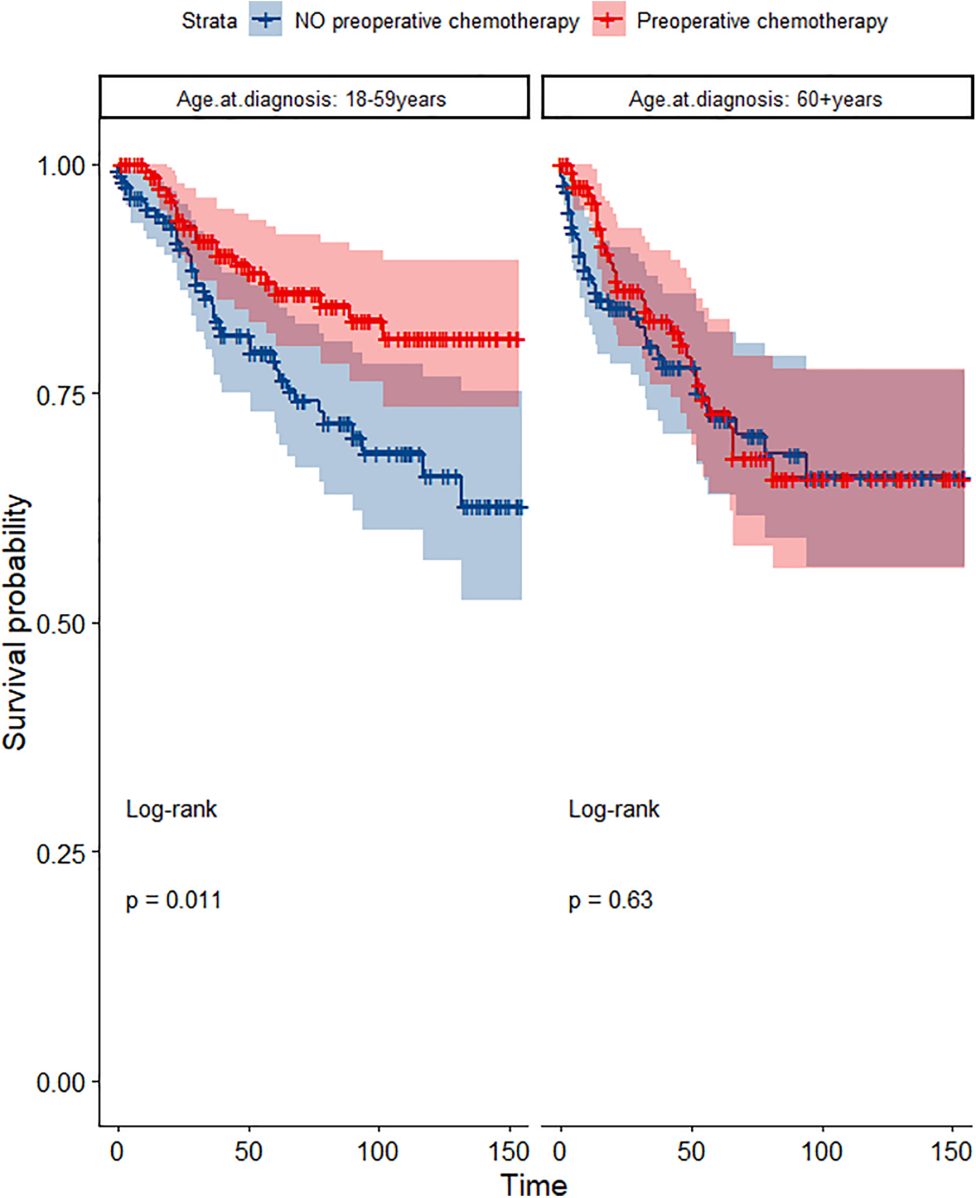
Survival curves of CSS in the POCT and no POCT groups in patients undergoing surgery for TETs in different age groups after propensity matching.

**Figure 11:**
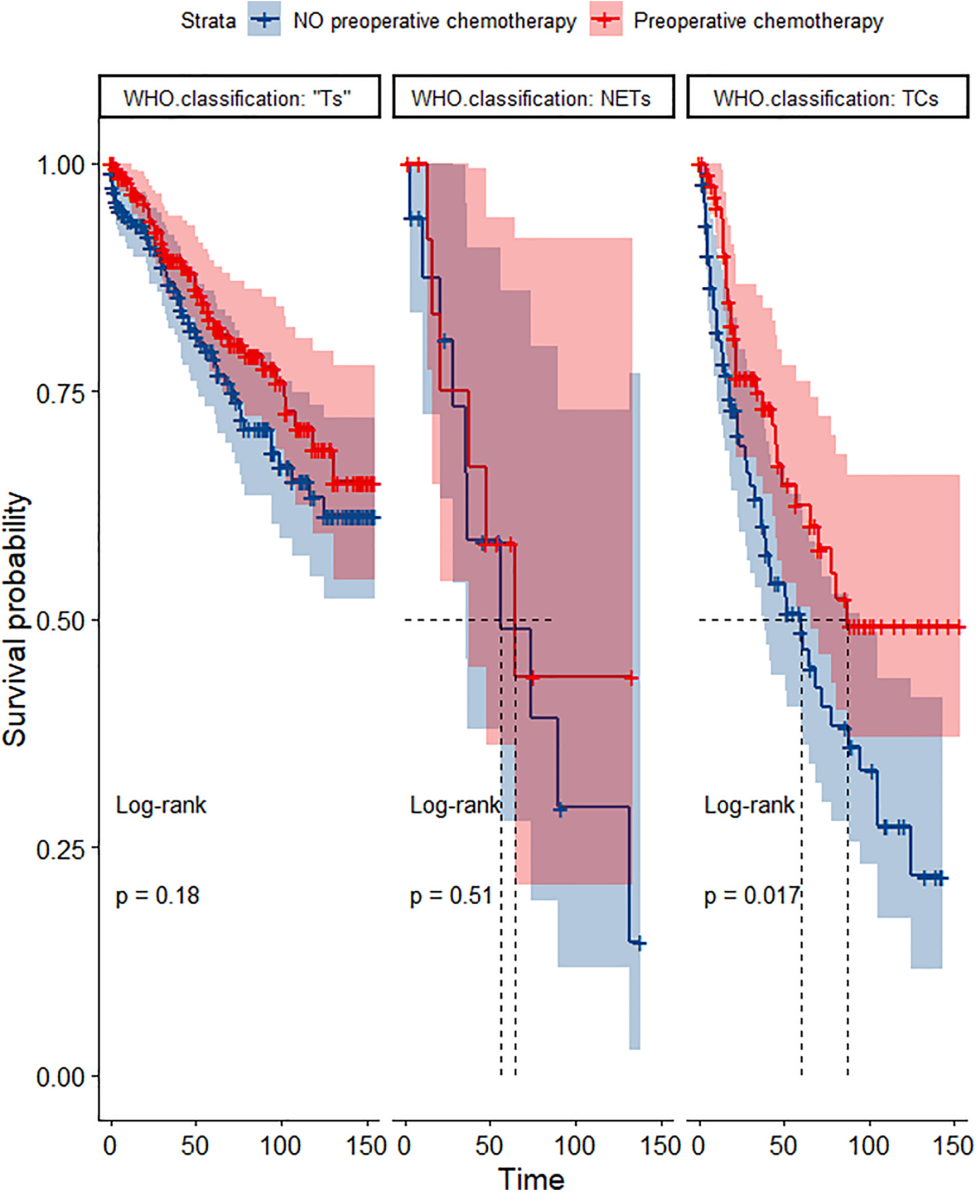
Survival curves of OS in the POCT and no POCT groups in patients undergoing surgery for TETs in different wHO classification groups after propensity matching.

**Figure 12:**
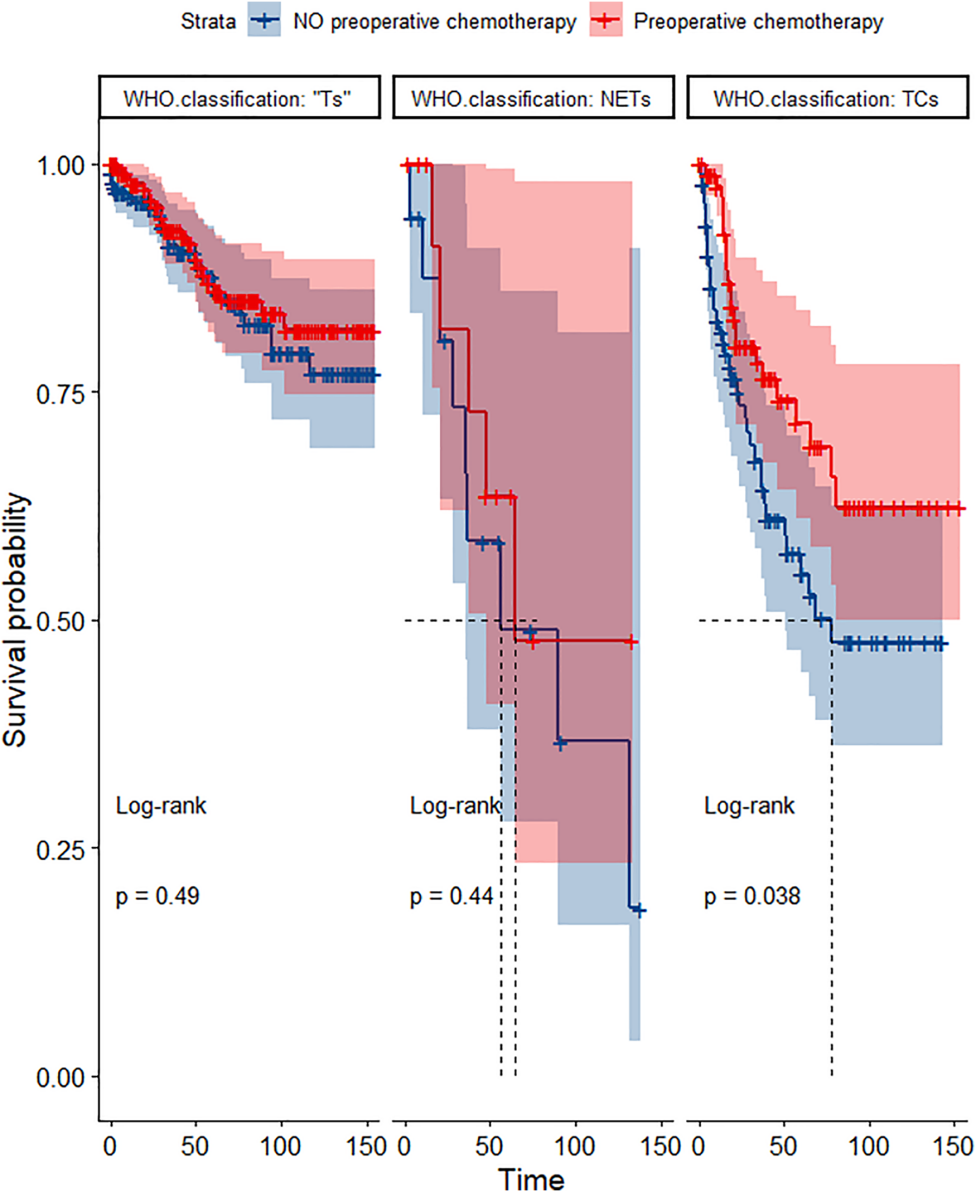
Survival curves of CSS in the POCT and no POCT groups in patients undergoing surgery for TETs in different WHO classification groups after propensity matching.

**Figure 13:**
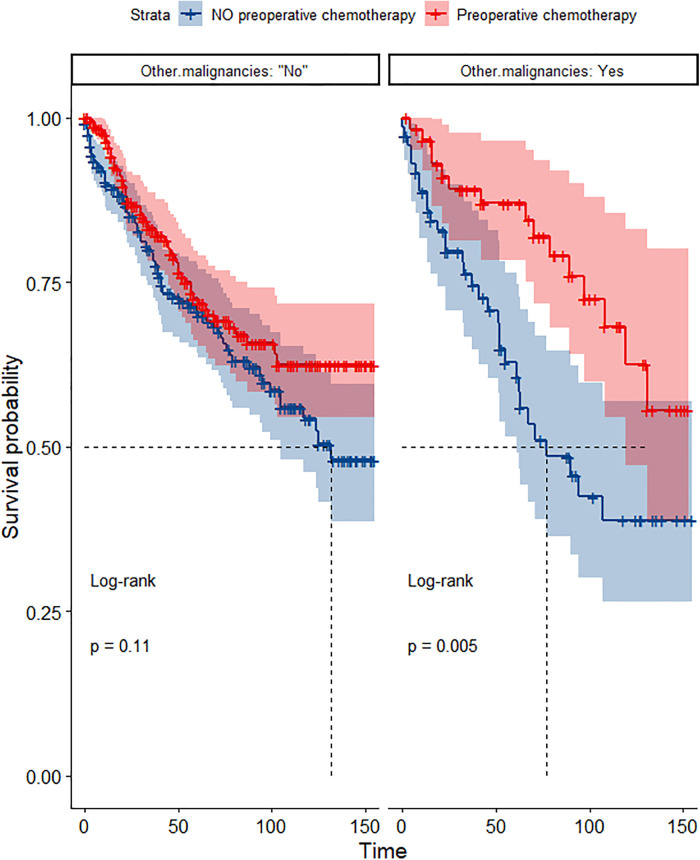
Survival curves of OS in the POCT and no POCT groups in patients with or without other malignancies undergoing surgery for TETs after propensity matching.

**Figure 14:**
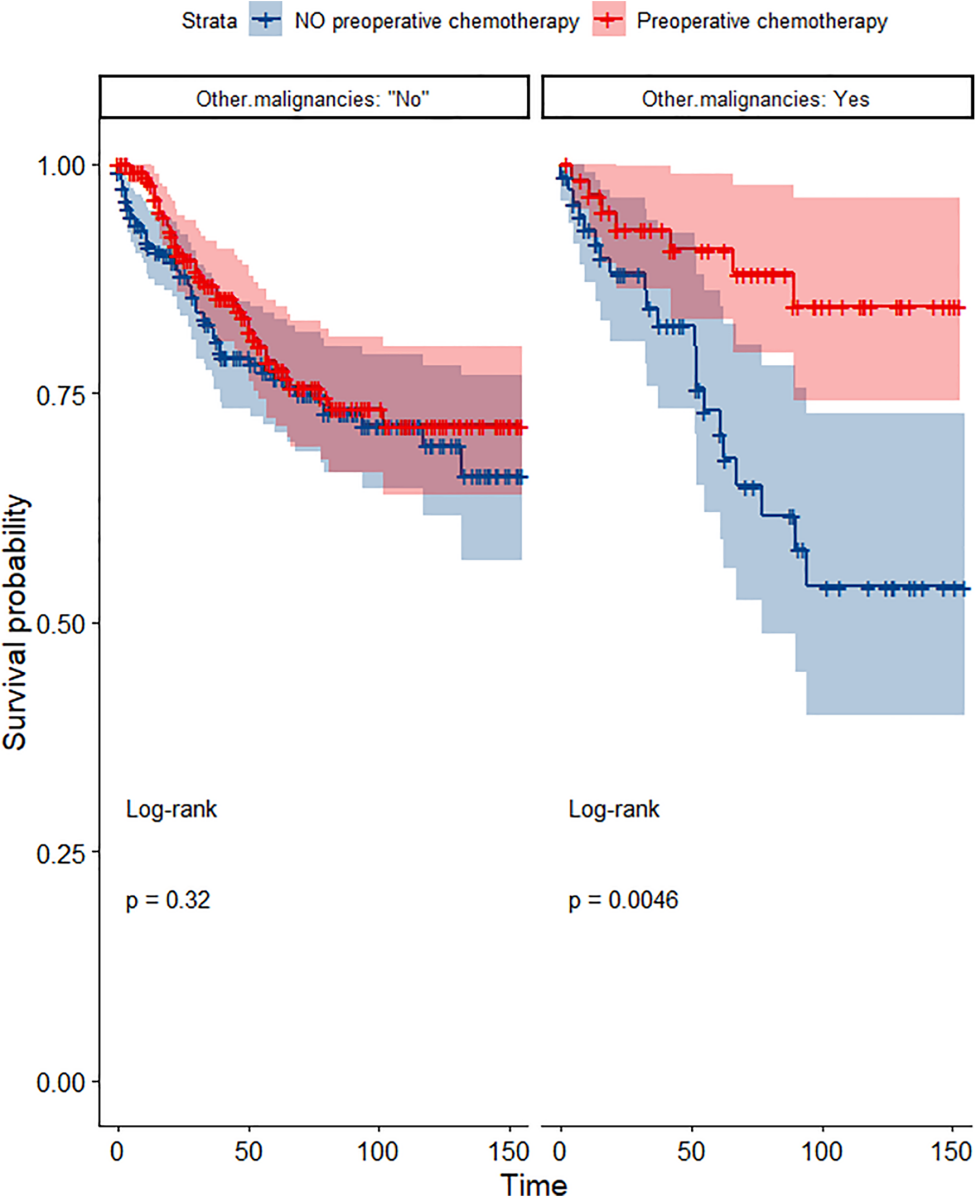
Survival curves of CSS in the POCT and no POCT groups in patients with or without other malignancies undergoing surgery for TETs after propensity matching.

In the propensity score-matched cohort, the overall survival and cancer-specific survival curves for the preoperative chemotherapy and no-preoperative chemotherapy groups are shown in Figures 5, 6. Preoperative chemotherapy significantly improves OS (Median survival times in months, 114.85 vs. 100.26, *P *= 0.0067) and CSS (Median survival times in months, 126.51 vs. 115.76, *P* = 0.031) in patients with TETs. In the subgroup analysis based on Masaoka–Koga staging, hazard ratios and 95% CIs for OS and CSS are shown in Figure 7, Overall survival and cancer-specific survival curves for the preoperative chemotherapy and no-preoperative chemotherapy groups are shown in Figures 8A,B. We observed that preoperative chemotherapy was a favorable factor for OS (HR: 0.45, 95%CI: 0.28–0.71) and CSS (HR: 0.49, 95%CI: 0.29–0.82) in patients with stage III/IV TETs, and preoperative chemotherapy significantly improved OS (Median survival times in months, 107.92 vs. 73.69, *P *= 0.00039) and CSS (Median survival times in months, 114.86 vs. 89.21, *P *= 0.0059) in stage III/IV patients, but the efficacy in stage I/IIA or IIB patients was not significant. In addition, subgroup analysis based on age, WHO staging, and presence of other cancers in Figure 7 showed that in terms of age, preoperative chemotherapy was a protective factor for OS (HR: 0.53, 95%CI: 0.33–0.84) and CSS (HR: 0.51, 95%CI: 0.30–0.87) in patients younger than 60 years of age with TETs, significantly improving OS (median survival times in months, 128.34 vs. 109.19, *P *= 0.0059, Figure 9) and CSS (median survival times in months, 134.69 vs. 118.00, *P *= 0.011, Figure 10) in this group of patients, but not in older patients; In terms of WHO-classification, preoperative chemotherapy was a protective factor for OS (HR: 0.58, 95%CI: 0.37–0.91) and CSS (HR: 0.58, 95%CI: 0.34–0.98) in patients with thymic carcinoma and significantly improved OS (Median survival times in months, 95.67 vs. 69.86, *P *= 0.017, Figure 11) and CSS (median survival times in months, 109.45 vs. 88.19, *P *= 0.038, Figure 12) in these patients; And in terms of the presence of other tumors, preoperative chemotherapy was a protective factor for OS (HR: 0.42, 95%CI: 0.23–0.79) and CSS (HR: 0.31, 95%CI: 0.13–0.73) in patients with TETs with multiple cancers and significantly improved OS (Median survival times in months, 121.13 vs. 89.00, *P *= 0.005, Figure 13) and CSS (median survival times in months, 137.76 vs. 106.32, *P *= 0.0046, Figure 14) in these patients.

## 4. Discussion

In this population-based study, we use data from the SEER database to evaluate the survival outcomes of 2,451 patients with TETs over the past 10-plus years. Compared to patients without preoperative chemotherapy, preoperative chemotherapy significantly improved OS in patients with stage III/IV TETs and did not significantly improve OS and CSS in stage I/IIA or IIB patients with TETs, both before and after propensity score matching. As a systemic treatment with some toxicities, preoperative chemotherapy significantly improved OS and CSS in younger patients or patients with multiple cancers with TETs. Therefore, we prefer to apply preoperative chemotherapy to advanced TETs detected by imaging, and patient history and physical condition should be carefully considered when applying chemotherapy.

Whether chemotherapy can improve the prognosis of patients undergoing surgery for TETs has been controversial. A multicenter analysis in Japan reveals that chemotherapy did not provide any survival advantage for patients with completely resected stage III and IV thymoma and thymic carcinoma (19), but chemotherapy in the study was limited to postoperative chemotherapy. Wei et al. reported a higher 5-year OS rate in patients with thymoma or thymic carcinoma who underwent direct surgery compared with those who received preoperative chemotherapy (20). In contrast, a study by Lucchi M et al. noted that patients who underwent surgery after neoadjuvant chemotherapy had better OS compared with patients who underwent primary surgery (21). Studies by Venuta F et al. and Macchiarini P et al. also reported a significant survival advantage after preoperative chemotherapy (22, 23).

In our study population, the median tumor size of 94.01 mm in patients who opted for preoperative chemotherapy was greater than the median of 66.19 mm in the group without preoperative chemotherapy, implying that preoperative chemotherapy was more frequently administered in patients with larger tumors. Early case reports and some small review studies have also shown that chemo can help to reduce tumor size and relieve symptoms (24). In the study by Federico Venuta et al. it was also suggested that preoperative chemotherapy had a down-staging effect (22). In the study by Macchiarini et al. in which preoperative chemotherapy was administered to seven patients with invasive thymoma, all patients had at least a 50% reduction in tumor size (23). Almost all research has concluded that R0 resection is a determinant factor associated with thymic tumor survival (19, 25, 26), but advanced thymic epithelial tumors often invade the lung parenchyma, heart and large vessel tumors making R0 resection difficult. The high chemosensitivity of thymic epithelial tumors (27) makes it possible to use use preoperative chemotherapy to improve R0 resection rates resulting in better OS and CSS. A phase II study by Kim et al. demonstrated that preoperative induction chemotherapy optimized surgical resectability of TETs, which, along with surgery and postoperative radiotherapy, comprised multidisciplinary treatment, which prolonged life (28).

As first-line neoadjuvant therapy, the most popular chemotherapy regimens are platinum derivatives, mainly cisplatin with anthracyclines and/or etoposide, and they show good activity against both thymoma and thymic carcinoma, often with response rates above 50% (29). Carboplatin-paclitaxel is mainly recommended for thymic carcinoma. The main side effects reported for chemotherapy tend to be nausea and vomiting, bone marrow suppression, and cardiotoxicity of anthracyclines (28, 30). Elderly patients have more complications and poor physical condition (10), which might decrease the tolerance of chemotherapy, so whether and when to use chemotherapy should be carefully chosen based on the patient's medical condition. A retrospective study by Samina Park et al. (31) suggested that the surgical group after neoadjuvant chemotherapy showed significantly higher transfusion rates (*P* = 0.003) and longer operative times (*P* < 0.001), but there was no evidence that neoadjuvant chemotherapy reduced long-term survival in patients with thymic epithelial tumors. The study by Cameron D et al. also concluded that chemotherapy induction followed by surgical treatment followed by radiotherapy is safe and probably the best sequence of treatment for carefully screened patients with advanced thymoma (32).

In addition, in our study, although the proportion of thymic carcinomas was smaller compared to thymomas, it appeared that thymic carcinomas responded more to induction chemotherapy, which is consistent with the findings of Robert et al. (27). Preoperative chemotherapy had several advantages over postoperative chemotherapy. For example, to prevent local tumor spread during surgery, to reduce tumor staging and thus improve surgical resection rates (33). Compared to postoperative chemotherapy, preoperative chemotherapy is better tolerated and most patients are able to reach surgery in good health, which also helps to improve the clinical status of the patient and to relieve symptoms (including myasthenia gravis remission) (24).

Previously, several SEER-based studies investigated the prognostic value of different treatment regimens for patients with TETs. However, we differ from previous studies by (1) using the SEER database for the first time to investigate the efficacy of preoperative chemotherapy in patients with thymic epithelial tumors, (2) performing propensity score matching to increase comparability and reduce bias at baseline, and (3) performing subgroup analysis by factors such as Masaoka–Koga staging to find the characteristics of patients with TETs suitable for preoperative chemotherapy.

The present study, like other SEER-based studies, has several limitations. First, although there is a wealth of data in the SEER database, it is not comprehensive and it lacks information on several important demographics, clinically relevant variables, and treatment modalities. For example, details of preoperative chemotherapy (including total dose of chemotherapy, daily fractions, and type of chemotherapeutic agent), whether the surgical margins were positive, and the patient's medical history and comorbidities. The lack of these variables leads to an incomplete clinical picture and potential bias, which may limit our assessment of preoperative chemotherapy. For this reason, we used the available data to focus on the impact of preoperative chemotherapy on long-term survival (≥1 month) in patients with thymic epithelial tumors. Second, although systemic treatment variables report the sequence of surgery and chemotherapy, they do not take into account the timing of events. Thus, it is possible that chemotherapy was administered more than 6 months prior to surgery or only 6 days prior to surgery. Chemotherapy administered prior to surgery may not have had sufficient time to anticipate the associated tumor response, which may underestimate the effect of preoperative chemotherapy. Although the SEER database is a representative national cancer registry with outstanding reliability and reproducibility of data collection and reporting procedures, and we used propensity score matching to minimize selection bias in preoperative chemotherapy, we could not completely exclude unmeasured or unpredictable confounding factors.

## 5. Conclusions

This study found that preoperative chemotherapy is a viable option for advanced thymoma with favorable overall and cancer-specific survival rates, but patient history and physical condition should be fully considered in conjunction with diagnostic imaging findings to assess patient tolerance to chemotherapy. Patients with thymic or multiple cancers may benefit from preoperative chemotherapy.

## Data Availability

The original contributions presented in the study are included in the article/**Supplementary Materials**, further inquiries can be directed to the corresponding author.
